# Effects of soft tissue surgery on transverse kinematics in patients with cerebral palsy

**DOI:** 10.1186/s12891-019-2955-8

**Published:** 2019-11-27

**Authors:** Byeong-Seop Park, Chin Youb Chung, Moon Seok Park, Kyoung Min Lee, Seong Hee Cho, Ki Hyuk Sung

**Affiliations:** 1 Department of Orthopaedic Surgery, Seoul National University Bundang Hospital, 82 Gumi-ro 173 Beon-gil, Bundang-Gu, Sungnam, Gyeonggi 13620 Republic of Korea; 2 Department of Orthopaedic Surgery, Gyeongsang National University Hospital, Jinju, Republic of Korea

**Keywords:** Soft tissue surgery, Cerebral palsy, Gait analysis, Transverse kinematics, Multilevel surgery

## Abstract

**Background:**

Gait disturbances, including flexed knee gait, stiff knee gait, and tip-toeing gait, are common in patients with cerebral palsy (CP). There has been no reports regarding kinematic changes in the transverse plane after soft tissue surgeries, such as distal hamstring lengthening (DHL), rectus femoris transfer (RFT), and tendo-Achilles lengthening (TAL). This study aimed to evaluate changes in the transverse plane after soft tissue surgery in patients with CP by assessing the effects of the DHL, RFT, and TAL.

**Methods:**

The study enrolled 156 consecutive patients (mean age, 8.4 years; range, 4.4 to 20.9), representing 213 operated limbs, who underwent soft tissue surgery including DHL with semitendinosus transfer, RFT, and TAL. All patients were assessed by preoperative and 1-year postoperative three-dimensional gait analysis. Changes in transverse plane kinematics after soft tissue surgery and affecting factors were analyzed.

**Results:**

Sagittal kinematics including knee flexion at initial contact, ankle dorsiflexion at initial contact, and mean ankle dorsiflexion in the stance phase were significantly improved after single event multilevel surgery (all *p* < 0.001). Transverse kinematics, including mean tibial rotation and foot progression angle, were significantly improved to a more external angle after soft tissue surgeries (− 2.9°, *p* = 0.004 and − 9.5°, *p* < 0.001). The mean hip rotation was significantly improved to a more external angle by RFT (− 4.7°, *p* = 0.010) and the foot progression angle was significantly improved to a more external angle by TAL (− 3.9°, *p* = 0.028).

**Conclusions:**

This study found that the transverse kinematics were improved to a more external angle after soft tissue surgery in patients with CP. Therefore, clinicians should consider that soft tissue surgery can affect the transverse plane kinematics in patients with CP. To confirm our findings, further research regarding the natural history of femoral and tibial torsion in children with CP is needed.

## Background

Gait disturbances, including flexed knee gait, stiff knee gait, and tip-toeing gait, are common in patients with cerebral palsy (CP) [1–3]. Flexed knee gait is characterized by an abnormally high knee flexion, and hamstring spasticity with contracture has been known as one of the main factors [4–7]. Stiff knee gait presents as delayed and/or reduced peak knee flexion during the swing phase [8, 9] and is considered to be caused by spasticity of the rectus femoris muscle. Tip-toeing gait, or equinus deformity of the ankle, is defined as a limitation of passive dorsiflexion beyond the neutral position, considered to be caused by spastic or contracted calf muscles [7, 10]. Several surgical procedures have attempted to resolve these abnormalities; distal hamstring lengthening (DHL), rectus femoris transfer (RFT), and tendo-Achilles lengthening (TAL) are commonly performed as part of a single event multilevel surgery (SEMLS) [11–13].

Transverse plane deformities, such as, in-toeing gait due to excessive femoral anteversion or internal tibial torsion, are also common in patients with CP and can be corrected by derotational osteotomy of the femur or tibia [5, 14–16]. Foot deformities including cavovarus or planovalgus deformity also contribute to problems in the transverse plane and can be corrected by corrective osteotomy and/or tendon surgery [14].

Several studies have reported improved motion in the sagittal plane after DHL, RFT, and TAL [3, 12, 13, 17–21]. However, to our knowledge, there have been few studies on changes in transverse plane kinematics after soft tissue surgery. Furthermore, transverse plane kinematics have not been frequently reported because of the increased variability of joint motion angle in the transverse plane due to differences in marker placement [22, 23] and soft tissue artifact at the thigh [24, 25]. Chong et al. [26] reported that the medial hamstring plays an important role in in-toeing gait, and Steinwender et al. [27] reported that soft tissue surgery that included multilevel medial lengthening of this muscle improved the internal rotation of the hip. This finding was supported by the results obtained by Jung et al. [28], who reported that pelvic retraction and hip internal rotation improved following soft tissue surgery.

Considering this, we hypothesized that DHL, RFT, and TAL can affect kinematics in the transverse plane as well as in the sagittal plane. There are no reports available regarding kinematic changes in the transverse plane for parts of the lower extremities, except for the hip and pelvis, following soft tissue surgery such as DHL, RFT, or TAL. Therefore, we performed this study to evaluate the changes in transverse kinematics after soft tissue surgery—namely, DHL, RFT, or TAL—using three-dimensional (3D) gait analysis.

## Methods

This retrospective study was approved by the institutional review board at our institute (a tertiary referral center for CP and the need for informed consent was waived.

Inclusion criteria were as follows: (1) consecutive ambulatory patients with CP who underwent SEMLS including DHL with semitendinosus transfer (STT), RFT, or TAL; and (2) patients for whom preoperative and 1-year postoperative 3D gait analysis were carried out. Exclusion criteria were as follows: (1) patients who underwent concomitant surgery which could affect transverse kinematics, such as femoral derotation osteotomy, tibial derotation osteotomy, or foot surgery (calcaneal lengthening osteotomy, tibialis posterior tendon split transfer, tibialis anterior tendon split transfer, Dwyer osteotomy, tibialis posterior tendon lengthening, triple arthrodesis, or triple osteotomy), 2) patients with a history of gait-correcting surgery or selective dorsal rhizotomy, and (3) patients who had incomplete or missing 3D gait analysis data.

From the medical record review, patients’ demographics including sex, age at surgery, gross motor function classification system (GMFCS) level, anatomical type of CP and type of concomitant surgeries, was obtained.

### Operative protocol

DHL with STT, RFT, and TAL were performed as part of a SEMLS by a single pediatric orthopedic surgeon (CYC). Preoperative 3D gait analysis and physical examination were used to plan the surgical procedures. The indications for DHL were an increased popliteal angle and increased knee flexion at initial contact or terminal swing. The procedure of DHL consists of gracilis lengthening, aponeurotic lengthening of the semimembranosus, and STT to the adductor magnus [13]. The procedure of RFT consists of transfer of the rectus femoris tendon to gracilis or sartorius, which is indicated by decreased or delayed peak knee flexion in the swing phase and a positive Duncan-Ely test [17]. The indications for TAL were fixed equinus deformity with negative Silfverskiold test and increased plantar flexion in stance phase. This procedure was performed using a coronal Z-plasty technique [11].

Following surgery, all patients underwent 4 weeks of immobilization with a short leg cast and/or knee immobilizer. Subsequently, patients were referred to a local rehabilitation center for muscle-strengthening exercises and gait training.

### Acquisition of kinematic data

3D gait analysis was performed a few days before surgery and was repeated at a time more than 1 year after surgery using a Motion Analysis system (Motion Analysis Corporation, CA, USA) equipped with ten cameras and two force plates. Markers were placed according to the Helen Hayes marker set [21] by two operators. Kinematic data were recorded as patients walked barefoot on a 9 m walkway [18, 21]. Data were recorded in triplicate and the averages calculated to determine the values of index variables. Preoperative and postoperative kinematic variables were compared in order to determine the effect of SEMLS including DHL with STT, RFT, or TAL. Transverse kinematics including mean pelvic rotation, mean hip rotation and mean tibial rotation over the gait cycle, and mean foot progression angle in stance phase, were considered outcome measures; as were sagittal kinematics, which included knee flexion at initial contact, minimum knee flexion in stance phase, mean ankle dorsiflexion in stance phase and ankle dorsiflexion at initial contact. Hip rotation was calculated from the relative motion between a distal medial-lateral axis of the thigh and a medial-lateral axis of the pelvis as viewed by an observer (on the pelvis) looking down the long axis of the thigh. Tibial rotation was calculated from the relative motion between a distal medial-lateral axis of the shank and a medial-lateral axis of the thigh as viewed by an observer (on the thigh) looking down the long axis of the shank. Foot progression angle was an absolute angle of the relationship between the long axis of the foot as defined by the foot marker placement and the direction of progression.

### Statistical analysis

Descriptive statistics such as the mean and standard deviation were used to summarize patient demographics. The Kolmogorov-Smirnov test was used to verify the normality of the distribution of variables. The Paired t-test was used to evaluate differences between preoperative and postoperative kinematics.

Bilateral limbs were included in this study; thus, a linear mixed model (LMM) was used to ensure statistical independence [29]. Postoperative changes in gait kinematics were adjusted for multiple factors using the LMM; with GMFCS level, type of involvement, DHL with STT, RFT, and TAL as the fixed-effect model; and follow-up duration and each subject as the random effect model.

Statistical analyses were carried out using R version 3.2.5 (R Foundation for Statistical Computing, Vienna, Austria). All tests were two-tailed, and *p*-values < 0.05 were considered statistically significant.

## Results

Between 2003 and 2016, preoperative and postoperative 3D gait analysis was carried out on 430 patients (706 limbs) who underwent SEMLS. After implementation of the inclusion and exclusion criteria, 156 patients (213 limbs) were finally enrolled in this study. The majority of patients were classified as GMFCS level I according to functional classification, and bilateral involvement according to type of involvement. The mean age at the time of surgery was 8.4 ± 3.2 years (range, 4.4 to 20.9). Average interval between preoperative and postoperative 3D gait analysis was 1.2 ± 0.8 years. The total number of surgical procedures was 507 (2.4 per limb) (Table 1).

**Table 1:** Patients demographics and summary of concomitant surgeries

	Value
Sex (male/female)	107/49
Laterality (right/left)	110/103
Type of involvement (unilateral/bilateral)	25/131
GMFCS level (I/II/III)	83/60/13
Age at surgery (years)	8.4 ± 3.2 (4.4 to 20.9)
Follow-up duration (years)	1.2 ± 0.8 (1.0 to 3.4)
Concomitant surgery	Limbs
Distal hamstring lengthening	199 (93.4%)
Tendo-Achilles lengthening	154 (72.3%)
Intra-muscular psoas lengthening	24 (11.3%)
Rectus femoris transfer	130 (61.0%)
Adductor tenotomy	7 (3.3%)

*GMFCS* Gross motor function classification system

Sagittal kinematics including knee flexion at initial contact, ankle dorsiflexion at initial contact, and mean ankle dorsiflexion in the stance phase were significantly improved after SEMLS (*p* < 0.001 in all cases). Transverse kinematics, including the mean tibial rotation and mean foot progression angle, were improved to a more external angle after soft tissue surgery (− 2.9°, *p* = 0.004 and − 9.5°, *p* < 0.001, respectively) (Table 2).

**Table 2:** Preoperative and postoperative kinematic data

Parameters	Preoperative	Postoperative	*P*-value	Normal control
Mean pelvic rotation (°)^a^	0.2 ± 7.9	−0.5 ± 5.9	0.181	− 1.0 ± 2.1
Mean hip rotation (°)^a^	3.3 ± 11.8	3.7 ± 10.1	0.713	− 1.6 ± 7.7
Mean tibial rotation (°)^a^	−2.3 ± 11.1	− 5.2 ± 11.1	0.004	− 17.4 ± 8.9
Mean foot progression angle (°)^a^	− 1.7 ± 12.5	− 11.2 ± 9.9	< 0.001	− 10.6 ± 6.1
Knee flexion at initial contact(°)	29.8 ± 12.4	22.4 ± 9.6	< 0.001	8.5 ± 3.9
Minimum knee flexion in stance phase (°)	8.1 ± 14.2	6.6 ± 8.7	0.094	7.4 ± 3.5
Ankle dorsiflexion at initial contact (°)^b^	−4.5 ± 11.9	4.3 ± 7.8	< 0.001	3.2 ± 3.2
Mean ankle dorsiflexion in stance phase (°)	1.5 ± 12.9	10.1 ± 5.6	< 0.001	7.7 ± 1.8

Data are presented as mean ± standard deviation

^a^Negative value means external rotation

^b^Negative value means plantarflexion

**Fig. 1:**
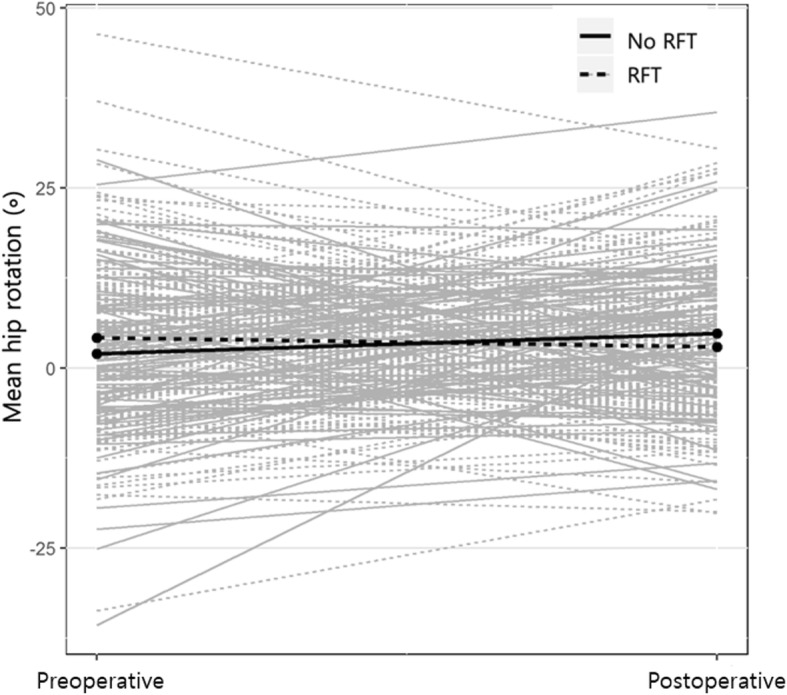
Change of mean hip rotation in patients who performed rectus femoris transfer (RFT) and those who did not perform RFT. The change of mean hip rotation in patients who underwent RFT was toward a more external rotation (4.7°) than those who did not undergo RFT

**Fig. 2:**
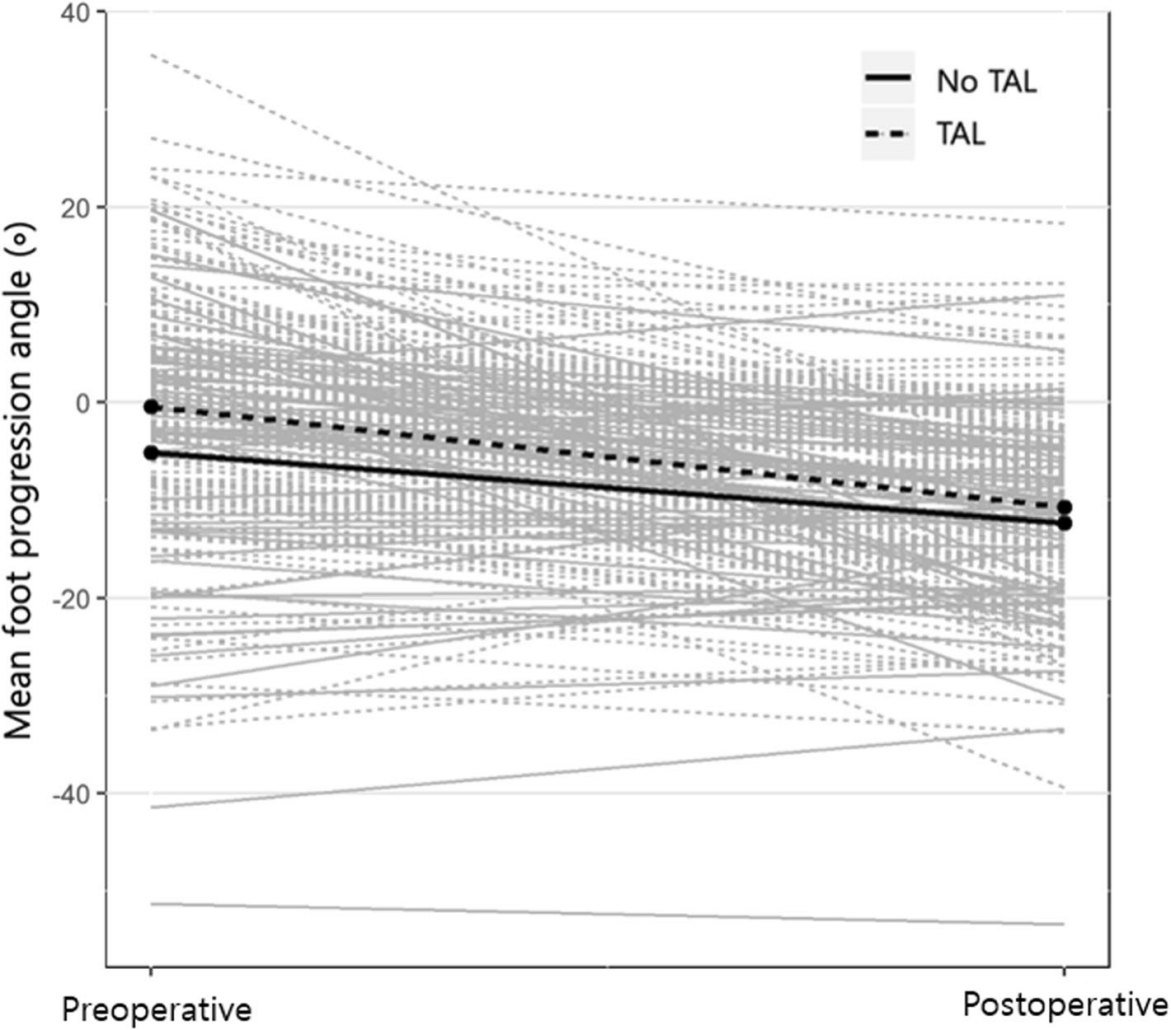
Change of mean foot progression angle in patients who performed tendo-Achilles lengthening (TAL) and those who did not perform TAL. The change of foot progression angle in patients who underwent TAL was toward a more external rotation (3.9°) than those who did not undergo TAL

After adjusting the changes in transverse kinematics for multiple factors, the mean hip rotation was significantly improved to a more external angle by RFT (*p* = 0.019), and the foot progression angle was also significantly improved to a more external angle by TAL (*p* = 0.028) (Table 3). The change in mean hip rotation in patients who underwent RFT was to a more external rotation (4.7°) compared to those who did not undergo RFT (Fig. 1). Similarly, the change in foot progression angle in patients who underwent TAL was to a more external rotation (3.9°) compared to those who did not undergo TAL (Fig. 2).

**Table 3:** Influencing factors for the change of transverse kinematics after surgery in patients with cerebral palsy

	Mean pelvic rotation (°)		Mean hip rotation (°)		Mean tibial rotation (°)		Foot progression angle (°)	
	Estimation (95% CI)	*p*-value	Estimation (95% CI)	*p*-value	Estimation (95% CI)	*p*-value	Estimation (95% CI)	*p*-value
DHL	−1.1 (−5.6, 3.5)	0.645	0.7 (−6.8, 8.1)	0.860	0.9 (− 7.5, 9.3)	0.833	−5.2 (− 11.7, 1.2)	0.112
RFT	2.0 (−0.4, 4.3)	0.104	−4.7 (−8.6, − 0.8)	0.019	1.8 (− 2.7, 6.3)	0.436	−1.2 (− 4.6, 2.3)	0.503
TAL	1.0 (−1.4, 3.4)	0.411	2.0 (−2.0, 5.9)	0.325	0.6 (−4.0, 5.2)	0.808	−3.9 (− 7.4, −0.4)	0.028
Type of involvement (bilateral)	− 3.4 (0.0, 11.3)	0.050	5.7 (0.0, 11.3)	0.048	−3.0 (− 9.3, 3.4)	0.365	−2.1 (−7.0, 2.8)	0.401
GMFCS level (I)								
II	0.2 (−2.1, 2.5)	0.864	−2.4 (−6.2, 1.4)	0.212	5.3 (0.7, 9.8)	0.023	0.0 (−3.4, 3.4)	0.986
III	−5.1 (− 9.2, −1.0)	0.014	0.1 (−6.6, 6.9)	0.970	7.8 (−0.2, 15.8)	0.056	−5.9 (− 11.9, 0.1)	0.055

*CI* Confidence interval, *DHL* Distal hamstring lengthening, *RFT* Rectus femoris transfer, *TAL* Tendo-Achilles lengthening, *GMFCS* Gross motor function classification system

## Discussion

Soft tissue surgeries including DHL with STT, RFT, and TAL are usually performed to correct abnormalities in the sagittal plane. Previous studies have reported good results following soft tissue surgery; however, kinematic changes in the transverse plane after soft tissue surgery remain unclear.

Initially, we hypothesized that kinematics in the transverse plane would be altered after DHL with STT, RFT, and TAL. Because all procedures target structures that affect the medial side of the lower leg, it is a reasonable assumption that the biomechanics will be different after these surgeries. The semitendinosus and gracilis are considered primary internal rotators of the tibia, and their points of insertion are located at the medial side of the tibia [30]. Therefore, reduction in ligament tightening following DHL alone or DHL with RFT would decrease the internal rotation torque. Furthermore, the pathway of the rectus femoris and its origin are located more laterally than those of the gracilis. If RFT is performed, the direction of force to the medial side of the knee will change from internal to external. We believe that this is why we observed altered hip and tibial rotation following DHL with STT and RFT.

In a study by Jung et al. [28], hip rotation after SEMLS became more external in CP patients with diplegia. The authors frequently performed DHL and RFT together, but the two operations were not separately analyzed. However, Lovejoy et al. [31] reported that hamstring lengthening caused decreased hip internal rotation, although not to the extent that an internal rotation gait pattern changed to an external rotation gait pattern. In addition, Arnold et al. [32] reported that the medial hamstring, adductor brevis, adductor longus, and gracilis were unlikely to substantially contribute to excessive internal rotation of the hip. For this reason, they suggested that surgical lengthening of the hamstrings or adductors is an inappropriate approach to reduce excessive internal rotation of the hip. Therefore, internal rotation of the hip cannot be changed to external rotation, even if DHL is performed. Overall, a rotational change in the transverse plane occurs after SEMLS, but the mechanisms behind this change are unknown. Our study showed that hip rotation in the transverse plane tends toward external rotation after RFT.

Lofterød et al. [33] reported that the foot progression angle became more external after SEMLS, although other factors did not have statistically significant relationships with SEMLS. Our study showed that not only the foot progression angle, but also tibial rotation became more external following SEMLS. However, while the above-mentioned study only considered patients with diplegia, we presented results for patients with both hemi- and diplegia obtained from a larger group (156 patients, 213 limbs). Hadley et al. [20] also reported that the foot progression angle changed toward a more external angle following multiple soft tissue release, although the reasons for this were unclear.

A previous study showed that the point of Achilles tendon insertion was approximately 2 cm from the midline to the medial side [34]. We believe that this may be the reason the foot progression angle became more external after TAL. In addition, the current study demonstrated that the change in foot progression angle was significantly associated with TAL. If the Achilles tendon is tightened, the calcaneus would rotate internally, causing other components of the foot to rotate internally along the calcaneus. Therefore, once the Achilles tendon was lengthened following TAL, the forces driving the internal rotation of the foot decreased.

There were some limitations in this study. First, we used the Helen Hayes marker set to perform 3D gait analysis. Knee rotation was calculated from the relative motion between the distal medial-lateral axis of the shank and medial-lateral axis of the thigh. However, we used the term “tibial rotation” instead of “knee rotation” because knee rotation is minimal and considered as tibial rotation in clinical practice. Therefore, the results of tibial rotation could be inaccurate if problems involving the knee were present. Second, several studies reported low repeatability in the transverse plane due to variabilities in the alignment of markers [22, 23]. However, markers were placed by a skillful operator for consistent identification of anatomical landmarks and positioning of markers in this study. Third, SEMLS is the treatment of choice for the correction of gait pattern in patients with CP. This poses significant challenges for the analysis of individual surgical methods such as DHL with STT, RFT, and TAL. Therefore, we have compensated for this restriction as much as possible through statistical methods. Fourth, a control group that consisted of patients with CP who had not undergone surgery was not included due to the retrospective design of this study. Bone remodeling occurs with age in children and can lead to changes in transverse kinematics. Two short-term longitudinal study showed remodeling of femoral anteversion in patients with CP who did not underwent femoral derotation osteotomy [15, 35]. However, several studies showed that remodeling of femoral anteversion did not occur during development in children with CP [36–38]. Therefore, long-term longitudinal study regarding the natural history of femoral and tibial torsion in CP is required.

## Conclusion

In conclusion, our study demonstrates that the transverse kinematics were significantly improved to a more external angle after soft tissue surgery in patients with CP. Therefore, clinicians should consider that soft tissue surgery can affect the transverse plane kinematics in patients with CP. However, appropriate osteotomy should be considered for the correction of transverse plane deformities because of the small degrees of change after soft tissue surgery.

## Data Availability

The data set supporting the conclusion of this article is available on request to the corresponding author.

## Abbreviations

CP: Cerebral palsy
DHL: Distal hamstring lengthening
GMFCS: Gross motor function classification system
LMM: Linear mixed model
RFT: Rectus femoris transfer
SEMLS: Single event multilevel surgery
TAL: Tendo-Achilles lengthening
